# Lactate induces PD-L1 in HRAS^G12V^-positive oropharyngeal squamous cell carcinoma

**DOI:** 10.18632/oncotarget.27348

**Published:** 2020-04-28

**Authors:** Alexander K. Verma, Shanta M. Messerli, W. Keith Miskimins

**Affiliations:** ^1^Cancer Biology and Immunotherapies Group, Sanford Research, Sioux Falls, SD, USA

**Keywords:** oropharyngeal cancer, lactate, Ras, anti-tumor immunity, PD-L1

## Abstract

Intratumoral lactate production negatively correlates with survival and tumor clearance in the setting of human papillomavirus positive oropharyngeal squamous cell carcinoma (HPV-positive OPSCC). Robust anti-tumor immune activity is required for tumor clearance in human patients and animal models of this disease, and intratumoral lactate interferes with this process. While lactate is known to directly inhibit T cell activity, recent evidence has demonstrated that lactate can affect gene expression in multiple cell types. We therefore sought to determine if lactate in the tumor microenvironment could aid immune evasion by inducing the expression of immune checkpoint co-inhibitors. Using a mouse cell line transformed with HPV16 E6, E7, and HRAS^G12V^, we determined that OPSCC cells carrying the HRAS^G12V^ mutant showed significantly increased expression of PD-L1 in the presence of extracellular lactate. Furthermore, we demonstrate here that lactate activates the MEK/ERK pathway in Ras-mutated cells.

## INTRODUCTION

The human papillomavirus (HPV) is responsible for 60–72% of oropharyngeal cancers (OPC) [1]. HPV positive tumors typically have a poor prognosis; patients have a 50% five-year survival rate following diagnosis [2]. In addition, these tumors often arise in a younger cohort than their HPV negative counterparts and patients often report significant comorbidities related to eating and speaking [3]. Because these tumors arise in close proximity to delicate structures in the head and neck, surgical excision and conventional radiation therapy can cause profound disfigurement and loss of quality of life. Finding effective targeted treatments can help prevent these nosocomial effects.

To study this cancer type we used a C57BL/6J murine oropharyngeal epithelial cell line transformed with HPV16 E6, E7, and HRAS^G12V^ (MEER cell model developed by Williams et al. [4]). This line is derived from oropharyngeal cells taken from a C57BL/6J mouse and transformed with HPV16 E6, E7, and HRAS^G12V^. Prior studies have established that intratumoral lactate levels negatively correlate with tumor clearance and animal survival in syngeneic mice implanted with MEER cells (Zhuang et al., Cancer Cell, Publication in progress). However, these trends were not observed in animals lacking mature B and T lymphocytes. Therefore, it was determined that lactate production by MEER cells inhibits a crucial anti-tumor immune response through an unknown mechanism. Other studies have established that an intact immune system is necessary for clearance of HPV-positive oropharyngeal squamous cell carcinoma (OPSCC) tumors [5].

Cancer cells of all types typically exhibit elevated rates of glycolysis even in the presence of sufficient oxygen for mitochondrial respiration, a phenomenon known as the Warburg effect [6]. Intratumoral lactate levels have been observed as high as 40 micromoles per gram of tissue in patient-derived tissue samples [7]. This change in glycolytic flux is thought to involve abnormally high expression of pyruvate dehydrogenase kinase and lactate dehydrogenase [8–10]. Recently, it has been appreciated that lactate can affect transcription patterns in cancer cells via numerous overlapping pathways [11]. Because OPSCC cells are frequently known to express immune checkpoint co-inhibitors, which downregulate the activity of tumor infiltrating lymphocytes [12], we sought to determine if lactate in the extracellular environment could induce the expression of immune checkpoint inhibitors in HPV-positive OPSCC.

## RESULTS

### Extracellular lactate induces PD-L1 expression in MEER cells

**Figure 1:**
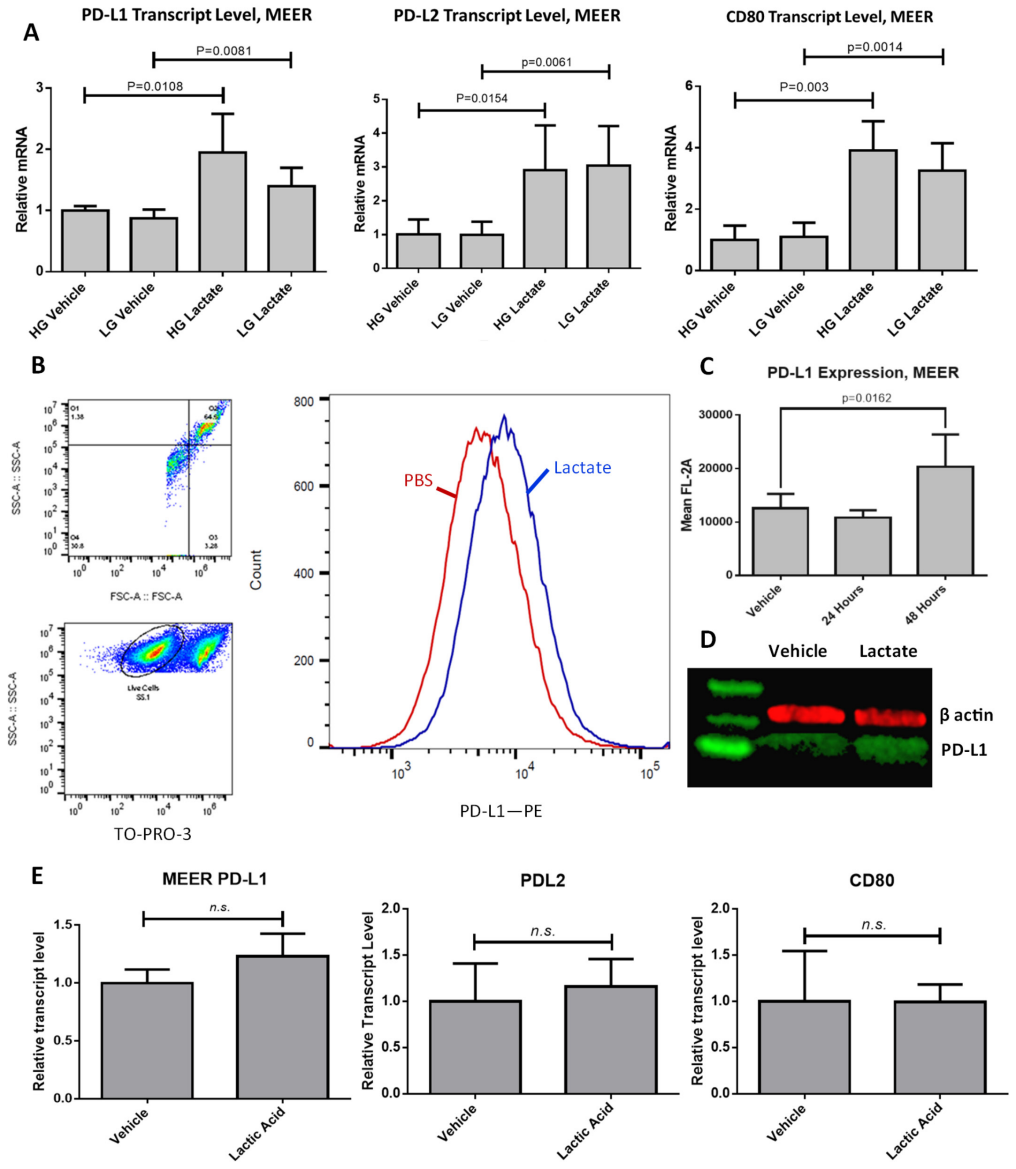
PD-L1 is upregulated in response to lactate exposure in MEER cells. (**A**) RT-qPCR results for MEER cells treated either with 10 mM lactate or an equivalent volume of PBS, in DMEM containing either 25 mM glucose (HG) or 2.5 mM glucose (LG) for 48 hours. (**B**) Gating strategy for flow cytometry and representative histogram of MEER cells treated with either 10 mM lactate (Blue) or PBS (Red) for 48 hours. Histogram height is normalized to the mode of samples tested. (**C**) Aggregate data of flow cytometry experiments. *N* = 8, 10,000 cells per sample. (**D**) Western blot of MEER cell lysate stained for PD-L1 (green) and β-actin (red). Cells were exposed to either PBS (left) or lactate (right) as described above for 48 hours. (**E**) RT-qPCR results for MEER cells treated either with 10 mM lactic acid or an equivalent volume of PBS.

To examine the effect of lactate on immune checkpoint coinhibitory expression we utilized the MEER cell line [4]. This is a mouse oropharyngeal epithelial cell line derived from oral scrapings of C57BL/6J mice and transformed with HPV16 E6 and E7, along with rat HRAS^G12V^ transfected on a pBABE-puro vector. We treated these cells with either 10 mM sodium lactate dissolved in phosphate buffered saline (PBS) or an equivalent volume of PBS as the vehicle treatment, in complete Dulbecco’s Modified Eagle Medium (DMEM) containing either 25 mM glucose (HG) or 2.5 mM glucose (LG). For RNA isolation these cells were treated for 4 hours or 24 hours. We observed that while no changes transcript level occurred at the 4 hour timepoint, at 24 hours we observed increased relative transcript levels of PD-L1 (1.945 times vehicle, *p* = 0.0108), PD-L2 (2.91 times vehicle, *p* = 0.0154), and CD80 (3.908 times vehicle, *p* = 0.003) (Figure 1A). Glucose concentration in the growth media did not have a significant effect on transcript levels. Surface expression of the PD-L1 protein as assessed by flow cytometry using non-permeabilized MEER cells did not increase by the 24 hour timepoint but did increase after 48 hours of exposure (1.618646 times vehicle, *p* = 0.0162) (Figure 1B–1D). Treatment with sodium lactate over this time period did not significantly alter media pH compared to vehicle (data not shown). These experiments were repeated in the presence of 10 mM lactic acid. This treatment did not increase transcript levels of PD-L1, PD-L2, or CD-80 (Figure 1E). We also tested the oropharyngeal squamous cell lines UPCI:SCC90 (HPV16-positive), UM-SCC47 (HPV16-positive), UM-SCC1 (HPV-negative), and UM-SCC84 (HPV-negative), as well as HeLa (HPV18-positive). We found that of these cell lines only UM-SCC90 showed increased PD-L1 expression in response to lactate (Supplementary Figure 1). However, in SCC90 cells we observe that a significant increase in PD-L1 levels at the cell surface occurs at 24 hours post treatment (Supplementary Figure 1A), which does not match the timescale observed in MEER cells. We also examined mouse oropharyngeal epithelial cells transfected with the LXSN vector (MOE LXSN) as a negative control. These cells showed a nonsignificant increase in PD-L1 transcript level in response to lactate (Supplementary Figure 1H).

### Lactate-induced PD-L1 does not depend on GPR81 in this cell model

**Figure 2:**
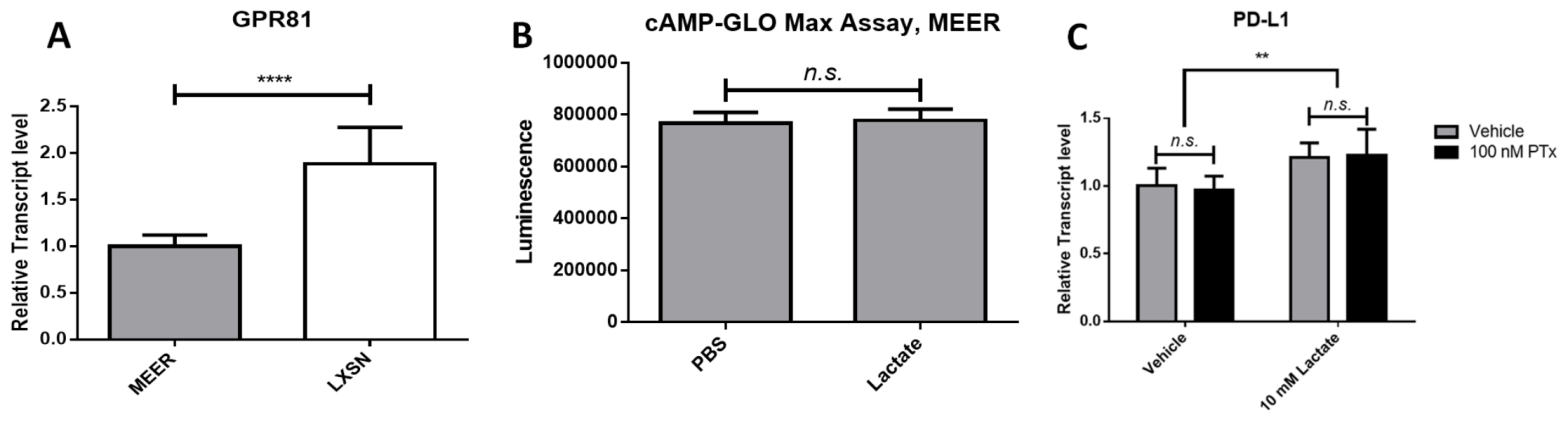
Lactate-induced PD-L1 in MEER cells does not depend on GPR81 signaling. (**A**) Relative transcript levels of GPR81 in MEER and MOE LXSN cells. (**B**) cAMP levels measured by a cAMP-GloMax assay in PBS or 10 mM lactate-treated MEER cells. (**C**) Relative transcript levels of PD-L1 in MEER cells treated with either 10 mM lactate or PBS and 100 nM PTX (black) or DMSO (gray). ^**^ *p* < 0.01, ^****^ *p* < 0.0001.

We next sought to determine if increased PD-L1 levels in response to lactate were mediated by GPR81, as has been shown in other cell models [13]. We compared transcript levels of GPR81 in both MEER (phenotype positive) and MOE LXSN (phenotype negative) cells. We found that GPR81 transcript levels were significantly higher in LXSN cells compared to MEER cells (1.887 times MEER, *p* < 0.00001) (Figure 2A). LXSN cells did not upregulate PD-L1 transcript levels in response to lactate (Supplementary Figure 1H). We next examined cyclic AMP (cAMP) levels in MEER cells treated either with 10 mM lactate or in PBS as described above using a cAMP-Glo Max assay (Promega). No significant difference was observed in cAMP levels between lactate-treated cells and vehicle-treated cells (Figure 2B). Finally, we examined PD-L1 transcript levels in MEER cells treated for 24 hours with lactate as described above in the presence of either 100 nM pertussis toxin (PTX) in dimethyl sulfoxide (DMSO) or an equivalent volume of DMSO. Previous studies of GPR81 have used this molecule to inhibit G-protein coupled receptors at the cell surface, including GPR81 [13–15]. The addition of PTX to cell treatments did not decrease PD-L1 transcript levels, nor did PTX treatment prevent a lactate-induced increase in PD-L1 transcript levels (Figure 2C).

### Overexpression of HRAS^G12V^ correlates with increased PD-L1 in response to lactate

**Figure 3:**
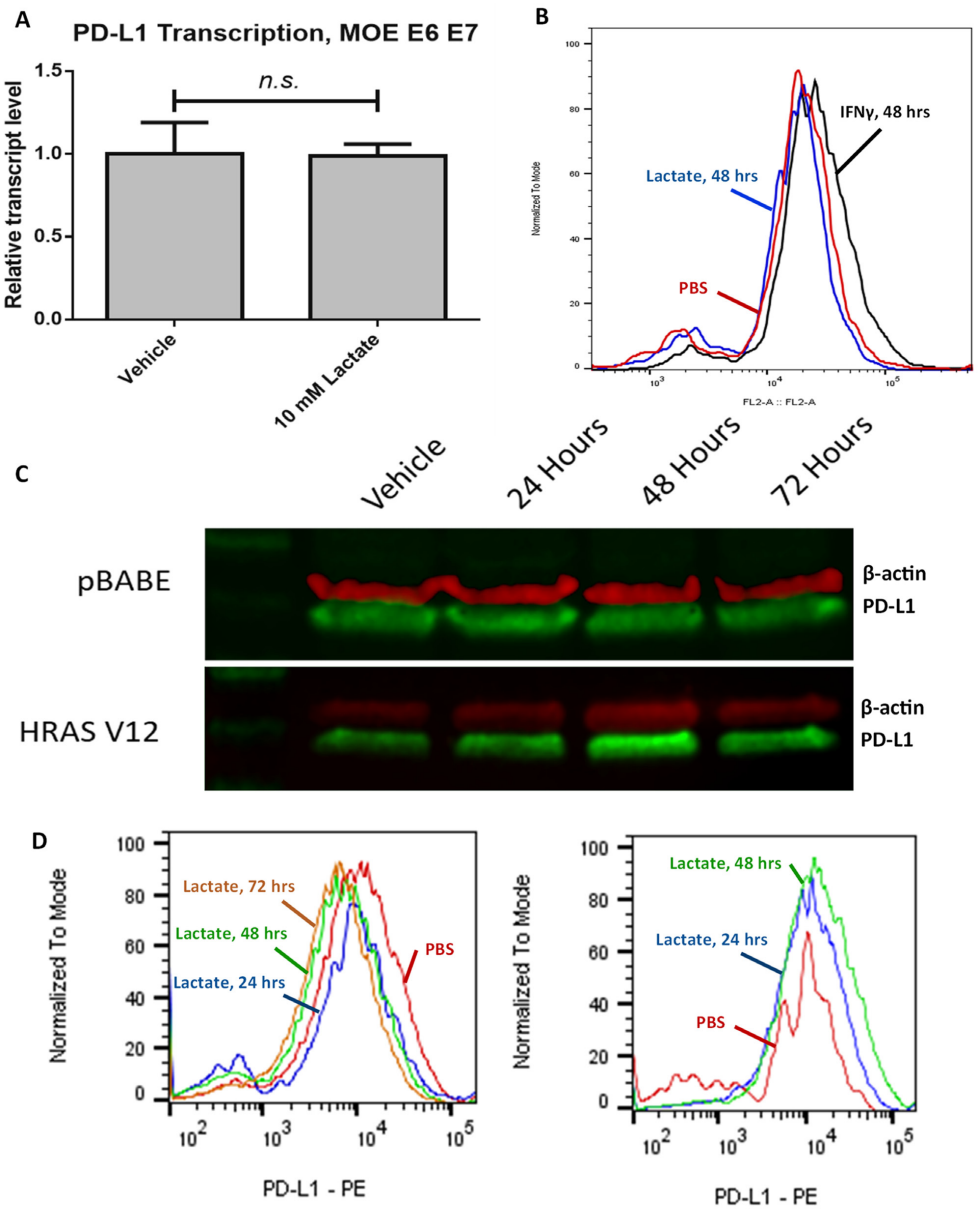
Lactate-induced expression of PD-L1 relies on HRAS^G12V^ expression. (**A**) RT-qPCR data for MOE E6E7 cells treated with either 10 mM lactate or an equivalent volume of PBS. (**B**) Flow cytometry analysis of MOE E6E7 cells treated with either PBS (red) or 10 mM lactate (blue), or with 100 ng/mL IFNγ (black) for 48 hours. Histogram height is normalized to the mode of samples tested. (**C**) Western blot of HEK293T cell lysate stained for PD-L1 (green) and β-actin (red). Cells were exposed to either PBS (left) or 10 mM lactate (right) over the indicated timeframe. Both pBABE-puro and HRAS^G12V^ transfected cells were analyzed in these experiments. (**D**) Flow cytometry analysis of HEK293T pBABE-puro (left) or HEK293T HRAS^G12V^ (right) cells treated with either PBS (Red) or 10 mM lactate for 24 (blue), 48 (green), or 72 (orange) hours. Histogram height is normalized to the mode of samples tested.

To elucidate which mutations are critical to the phenotype we have observed, we repeated the above tests using a mouse oropharyngeal epithelial cell line transfected with HPV E6 and E7 but lacking the HRAS^G12V^ mutation seen in the MEER cell line (MOE E6E7). We found that lactate exposure did not increase PD-L1 transcript level in this cell line after 24 hours (Figure 3A). After 48 hours exposure to lactate, we observed a small but significant increase in PD-L1 surface expression in MOE E6E7 cells (1.0935 times vehicle, *p* = 0.015692). However, 48 hour treatment with 100 ng/mL IFN-γ significantly increased PD-L1 expression by a much greater degree (1.334 times vehicle, *p* < 0.00001) (Figure 3B).

To determine the role of the HRAS^G12V^ mutation in mediating our observed phenotype, we repeated our previous experiments in HEK293T cells transfected either with HRAS^G12V^ or the empty pBABE puro vector. We found that HEK293T pBABE cells do not show an increase in surface expression of PD-L1 even after 72 hours of exposure, while HEK293T HRAS^G12V^ cells demonstrated increased membranous PD-L1 following lactate exposure (1.344 times vehicle after 24 hours treatment, *p* = 0.159992; 1.6343 times vehicle after 48 hours treatment, *p* < 0.00001) (Figure 3C, 3D). We also observed that overall PD-L1 levels seemed to peak after 48 hours of lactate exposure in the HEK293T HRAS^G12V^ cells.

### Lactate activates the MEK/ERK pathway

**Figure 4:**
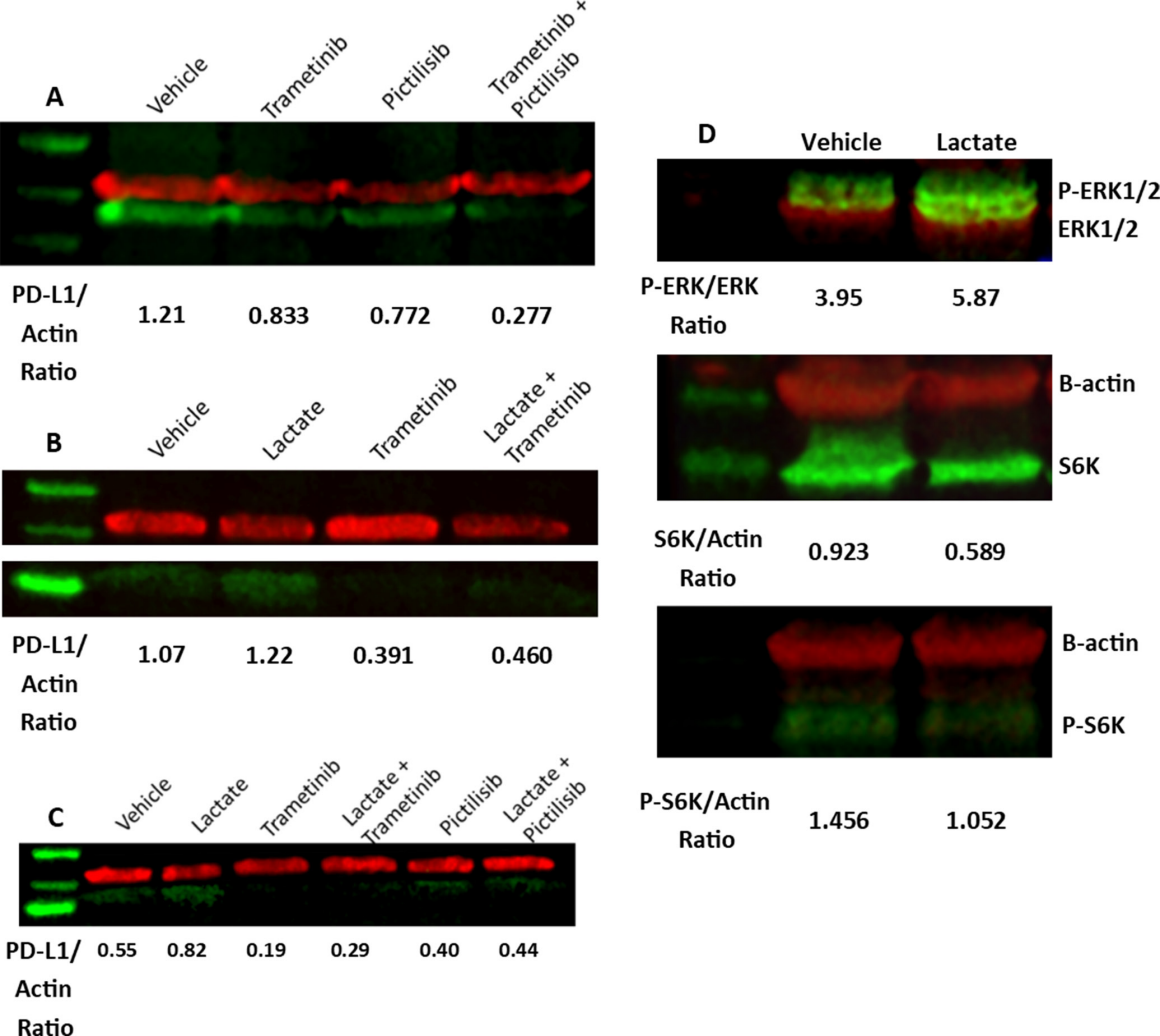
MEK activity contributes to PD-L1 expression. (**A**) MEER cells were exposed to DMSO, 25 nM Trametinib, 500 nM Pictilisib, or both for 24 hours. Western blot of cell lysate stained for PD-L1 (green) and β-actin (red). (**B**) MEER cells were exposed to PBS or 10 mM sodium lactate and 25 nM Trametinib or DMSO for 48 hours. Western blot of cell lysate stained for PD-L1 (green) and β-actin (red). (**C**) HEK293T HRAS^G12V^ cells were treated with DMSO, 25 nM Trametinib, or 500 nM Pictilisib in combination with either PBS or 10 mM sodium lactate for 48 hours. Western blot of cell lysate stained for PD-L1 (green) and β-actin (red). (**D**) Top: Western blot of MEER cell lysate stained for phosphoERK1-T202/Y204 & phosphoERK2-T185/Y187 (green) and pan-ERK (red). Middle: Western blot of MEER cell lysate stained for S6K (green) and β-actin (red). Bottom: Western blot of MEER cell lysate stained for phosphoS6K-T389 (green) and β-actin (red).

We next examined the effect of Ras signaling pathways on PD-L1 expression in our MEER and HEK293T HRAS^G12V^ cell models. To selectively inhibit phosphoinositol-3-kinase (PI3K) and MEK we used 500 nM Pictilisib and 25 nM Trametinib, respectively. MEER cells treated with either of these drugs for 24 hours showed decreased PD-L1 expression. A combination of both drugs had an additive effect, further decreasing PD-L1 levels (Figure 4A). MEER cells treated with Trametinib for 48 hours almost completely abrogated PD-L1 expression. However, treatment with this drug did not prevent an increase in PD-L1 expression in response to lactate (Figure 4B). This finding was also observed in HEK293T HRAS^G12V^ cells. HEK293T HRAS^G12V^ cells also demonstrated decreased PD-L1 levels in response to pictilisib treatment, though the decrease in PD-L1 expression was not as pronounced as with trametinib treatment (Figure 4C).

To determine if lactate could directly stimulate downstream elements of Ras or PI3K signaling, we assessed phosphorylation of ERK 1/2 and S6K in lactate-treated MEER cells. We observed an increase in phosphorylation of ERK1 at residues T202 and Y204 and ERK2 at residues T185 and Y187 following 48 hours of lactate exposure. S6K phosphorylation at T389, however, did not increase with lactate treatment (Figure 4D).

## DISCUSSION

Oropharyngeal cancer shows great potential for the application of immunological therapies. Recent empirical data demonstrates that patients may benefit from therapies that target immune checkpoint coinhibitors [16, 17]. To maximize the therapeutic potential of these treatments, further studies are needed to understand intratumoral signals that regulate immune checkpoint co-inhibitor expression.

In this publication we have examined how lactate, a common metabolic byproduct in the tumor microenvironment, affects expression of PD-L1 in a subset of human papillomavirus-positive oropharyngeal cancers. We find that lactate introduced into the extracellular environment causes an increase in transcript levels of PD-L1 and an increase in the transcript levels of other immune checkpoint co-inhibitors in a mouse model of HPV-positive OPSCC. PD-L1 levels were also increased at the cell surface in response to lactate treatment, as demonstrated using flow cytometry with non-permeabilized cells. This phenomenon was most likely not related to GPR81 activity. We further found that this phenotype can be replicated by the introduction of a mutated oncogenic Ras isoform, HRAS^G12V^, into the HPV-negative cancer cell line HEK293T. Mutations at this residue are common across all Ras isoforms and all cancer types and may predict a similar phenotype in other cancer lines [18]. Finally, we find that inhibition of signals downstream of Ras can decrease overall PD-L1 expression but does not completely prevent lactate from inducing expression. Furthermore, signs of MEK activity increase in response to lactate exposure but signs of mTOR activity do not.

These findings raise a number of questions about the physiological role of lactate in the tumor microenvironment and its effects on anti-tumor immunity. Lactate has been observed in tumor tissue at concentrations as high as 40 micromoles per gram [7]. In these cells the time of exposure necessary to induce changes to PD-L1 is significantly longer than would be expected with activation of a surface receptor [13]. This suggests that lactate influx into the cell via monocarboxylate transporter 1 (MCT1) might be a rate limiting step in inducing this phenotype. A similar effect has been observed in WiDr colon cancer cells, MDA-MB-231 triple-negative breast cancer cells, and TLT hepatocarcinoma cells, though these studies did not examine effects of lactate import on PD-L1 expression [19, 20]. Additional evidence is needed to determine if lactate import via MCT1 is a necessary step in the induction of PD-L1. It is also of interest to determine if the changes in immune checkpoint coinhibitory expression induced by extracellular lactate are of physiological consequence in the animal model.

Previous research on HPV-positive OPSCC indicates that the anti-tumor immune response plays a central role in tumor clearance and survival for this cancer. Conventional therapy with cisplatin and radiation (CRT) is found to induce a targeted immune response to HPV-positive tumors, which is necessary for tumor clearance in mouse models [5]. In human patients, the presence of HPV16-responsive CD8+ T cells enhanced patient survival following conventional therapy [21]. HPV-positive OPSCC tumors have one of the highest degrees of immune cell infiltration among all tumor types [22], indicating that these tumors are strongly immunogenic. Because of this immunogenic phenotype, immune checkpoint coinhibitory therapy holds some promise as an adjuvant to conventional chemotherapy and radiation. In a clinical trial of OPSCC patients with tumors refractory to cisplatin and cetuximab, approximately 50% of participants showed positive response to pembrolizumab, an antibody targeting the PD-1 receptor [23]. Other trials with antibodies targeting PD-1 have shown similar clinical response [17, 24–26]. However, these benefits are limited to a small subset of patients, as has been observed in other cancer types [27–30]. Furthermore, PD-L1 expression by tumor cells is not always predictive of patient response to PD-1/PD-L1 blockade [17, 31]. Because individual response to anti-PD-1 therapy is varied, it is of immense therapeutic value to identify molecular predictors of immunotherapy response.

Other studies have demonstrated that lactate can activate the orphan G-protein coupled receptor GPR81 [13]. These studies have used lactate in culture media at 10 mM, the same concentration we have used for our experiments However, we noted that GPR81 transcript levels did not correlate with the phenotype of increased PD-L1 in response to lactate. In comparing GPR81 transcript levels between MEER cells and MOE LXSN cells, we determined that transcript levels were higher in MOE LXSN cells than MEER cells (Figure 2A). We demonstrated that MOE LXSN cells did not significantly upregulate PD-L1 when cultured with lactate (Supplementary Figure 1H). Furthermore, we did not observe any changes in intracellular cAMP production following lactate treatment (Figure 2B). GPR81 is an inhibitory G protein specific to lactate that is frequently expressed in cancerous cells [32–34]. Activation of this receptor should lead to a decrease in intracellular cAMP [15, 35]. Finally, we demonstrated that treatment with PTX did not prevent a lactate-induced increase in PD-L1 transcript level (Figure 2C). Pertussis toxin specifically inhibits inhibitory G proteins by modifying a cysteine residue on the alpha subunit [36]. This toxin has been used to inhibit GPR81 in other studies [13, 34]. Taken together, the findings we have presented here indicate that lactate does not activate a G-protein coupled receptor in this cell model.

The Ras signaling pathway is commonly activated in cancers of all types, including oropharyngeal cancers [18, 37]. Previous experiments in mouse models have shown that both the PDZ binding motif of E6 and the oncogenic HRAS^G12V^ mutation are required for tumorigenesis [38]. This pathway shows extensive crosstalk with the PI3K-mTOR pathway [39], therefore it is important to determine if effects of oncogenic Ras signaling are due to the activity of the MEK/ERK pathway as opposed to other key pathways. Our findings show that lactate-treated cells carrying an oncogenic Ras mutation show increased phosphorylation of ERK1 and ERK2 at sites associated with MEK1/2 activity. However, S6K phosphorylation does not increase with lactate treatment. Taken together these findings indicate that the MEK/ERK pathway is responsible for translating the signal induced by lactate.

A key point of consideration with our findings is that, while inhibition of MEK activity with trametinib dramatically reduced PD-L1 expression in MEER cells and HEK293T HRAS^G12V^ cells after 48 hours, this treatment did not completely prevent a lactate-induced increase in PD-L1 (Figure 4B, 4C). This indicates that MEK activity increases baseline PD-L1 expression, as has been shown in other publications [40, 41], but that MEK activity alone does not enable a phenotype in which lactate enhances PD-L1 protein levels. Despite this, we see an increase in ERK phosphorylation at sites consistent with MEK activity (Figure 4D), indicating that lactate directly stimulates the Ras-Raf-MEK-ERK pathway. As S6K phosphorylation is not changed in response to lactate exposure, it seems that mTOR is not activated and therefore lactate does not stimulate the PI3K/mTOR pathway. Other studies have demonstrated that lactate can induce ERK phosphorylation at sites consistent with MEK activation [42, 43]. However, these studies have typically been done in myocytes and not cancer cells. A recent publication demonstrated that lactate in the tumor microenvironment can activate the ERK pathway in tumor associated macrophages, which is associated with tumor invasion and metastasis [44].

Extracellular lactate correlates with poor patient outcomes in the setting of oropharyngeal cancer. Because oropharyngeal squamous cell carcinoma, particularly human papillomavirus-positive tumors, are highly immunogenic and require host immune activity to clear tumors, the relationship between intratumoral lactate and anti-tumor immune activity is of importance in targeting therapies to these tumors. Here we show that lactate induces the expression of immune checkpoint coinhibitors in tumor cells carrying the oncogenic HRAS^G12V^ mutation. We further showed that lactate activates the MEK/ERK pathway but not the PI3K pathway. These findings indicate that intratumoral lactate may suppress tumor infiltrating lymphocytes by upregulating immune checkpoint coinhibitor expression. This indicates that in the treatment of HPV-positive OPSCC therapies that target the Ras-Raf-MEK-ERK pathway or those that reduce lactate production may improve patient outcomes.

## MATERIALS AND METHODS

### Materials

Sodium lactate was purchased from Sigma Chemical Company (Sigma, St. Louis, MO, USA). Trametinib (Mekinist/GSK1120212) and pictilisib (GDC-0941) were purchased from SelleckChem (Houston, TX, USA). Antibodies were obtained for β-actin (Sigma), PD-L1 (Invitrogen, CA, USA), pan-ERK (BD Biosciences, CA, USA), and Phospho-p44/42 MAPK (Erk1/2)(Thr202/Tyr204) (Cell Signaling Technology, MA, USA). R-phycoerythrin-conjugated Donkey-anti-rabbit secondary was purchased from Jackson Immunoresearch (West Grove, PA, USA). Fluorophore-conjugated anti-mouse and anti-rabbit antibodies for western blotting were obtained from LI-COR (Lincoln, NE, USA). pBABE puro H-Ras V12 was a gift from William Hahn (Addgene plasmid # 9051; http://n2t.net/addgene:9051; RRID:Addgene_9051). RT-qPCR primers were purchased from Integrated DNA Technologies (Skokie, IL, USA).

### Cell culture

MEER, MOE E6E7, and MOE LXSN cells were a gift from Paola Vermeer (Sioux Falls, SD, USA). HEK 293T, HeLa, UM-SCC1, UM-SCC47, UM-SCC84, UPCI:SCC90, 4T1, and B16-F10 cells were obtained from ATCC. Cell lines were cultured in Dulbecco’s Modified Eagle Medium with 10% Fetal Bovine Serum, 100 U/ml penicillin, 100 μg/ml streptomycin, and 500 ng/ml amphotericin B (Hyclone, Logan, UT, USA) in a humidified incubator at 37°C with 5% CO_2_. All cell lines were tested for mycoplasma infection.

### Stably transfected cell lines

Plasmid-containing E. coli were amplified in LB media with 100 μg/mL ampicillin for 24 hours at 37°C. Plasmid DNA was isolated using a Plasmid Midi Kit from QIAGEN (Germantown, MD, USA). 4 × 10^6^ HEK293T cells were cultured as described above in a 100 mm dish. After 24 hours incubation media was replaced with antibiotic-free DMEM with 10% FBS. Opti-MEM media (Hyclone) was used to combine 30 μg plasmid DNA with 60 μL lipofectamine 3000 (Invitrogen) according to the protocol given by Invitrogen and added to cells. Following 48 hours of incubation complete DMEM containing 2.5 μg/mL puromycin was added to cells. Individual colonies were selected and cultured as described above.

### RT-qPCR analysis

RNA was isolated from 1 × 10^6^ cells using a Maxwell 16 LEV simplyRNA Cells kit (Promega, Madison, WI, USA) according to the manufacturer’s protocol. RNA concentrations were assessed using a NanoDrop spectrophotometer (ThermoFisher). RNA quality was determined using an Agilent 2100 Bioanalyzer and Agilent RNA 6000 Pico kit (Santa Clara, CA, USA). For all samples RIN was 9 or greater. cDNA was transcribed from the template using a High Capacity cDNA Reverse Transcription kit (Applied Biosystems, Carlsbad, CA, USA) according to the manufacturer’s protocol. This cDNA was mixed with ABI SYBR Green Master Mix (Applied Biosystems), and the mixture was subjected to amplification using a Mx3005P Thermocycler (Agilent) according to the manufacturer’s protocol. Primers were used at a final concentration of 400 nM. Samples were heated to 95°C for 10 minutes. Subsequently, samples were subjected to 40 cycles of 30 seconds each at 95°C, 50°C, and 72°C, in that order. Finally, a melting curve was created by stepwise temperature change from 50°C to 95°C. Threshold fluorescence was determined automatically by MxPro qPCR software (Agilent). β-actin and HPRT1 were used as reference genes. Primer sequences are listed below in Table 1. Each sample was repeated in triplicate and analyzed using Livak method, with ΔΔCT normalized to the vehicle group.

**Table 1:** RT-qPCR primer sequences used

β-actin
Forward – 5′- GCCTTCCTTGGGTATG - 3′ Reverse – 5′- AGGAGCCAGAGCAGTAATCTC - 3′
CD80
Forward – 5′- TCCATCAAAGCTGACTTCTCT - 3′ Reverse – 5′- ATGCCAGGTAATTCTCTTCCATTT - 3′
GPR81
Forward – 5′- CATGAAGACCTGGAAGTCAA - 3′ Reverse – 5′- CAAGATGACCAAAGTCCAGA - 3′
HPRT1
Forward – 5′- GTCCCAGCGTCGTGATTAGC - 3′ Reverse – 5′- GGGACGCAGCAACTGACATT - 3′
PD-L1 - Mouse
Forward – 5′- CTGCTTGCGTTAGTGGTGTA - 3′ Reverse – 5′- GTTGCTGTGCTGAGGCTTAA - 3′
PD-L1 - Human
Forward – 5′- CACGGTTCCCAAGGACCTAT - 3′ Reverse – 5′- TGGAGGATGTGCCAGAGGTA - 3′
PD-L2
Forward – 5′- GATACCAGACAGCAGGACAGA - 3′ Reverse – 5′- GCAGACAAAGCCCCAAACA - 3′

### Western blotting

Whole-cell protein extracts were prepared from cell lines MEER and transfected HEK293T in RIPA buffer (Thermo) and 0.2 mM protease inhibitor cocktail (ThermoFisher). Protein concentration was determined using a Pierce BCA Protein assay kit (ThermoFisher). Cell lysates containing 30 μg protein were separated on a 4–20% gradient SDS-PAGE gel at 160 volts for 1 hour and transferred to a polyvinylidene fluoride (PVDF) membrane (Millipore, Billerica, MA, USA) using a BioRad Trans-Blot Turbo system (Hercules, CA, USA). The membrane was blocked in 0.1% tris buffered saline tween (TBST) with 10% blocking agent for 15 minutes at room temperature and incubated overnight at 4°C with primary antibody. After incubation with anti-rabbit and anti-mouse fluorophore conjugated antibodies followed by extensive washing, the bound antibodies were visualized using a LI-COR Odyssey system (Lincoln, NE, USA). Band intensity was quantified using LI-COR Image Studio. Western blots have been cropped and contrast-enhanced for clarity.

### Flow cytometry

Following treatment, cells were harvested using PBS with 4 mM ethylenediaminetetraacetic acid (EDTA). Cells were washed in phosphate buffered saline with 1% bovine serum albumin (Sigma), incubated with anti-PD-L1 antibody for 30 minutes, washed, incubated with secondary antibody for 30 minutes, washed twice, and analyzed using a BD Accuri C6 flow cytometer (Becton Dickinson, Franklin L, NJ, USA). Data was analyzed using FlowJo 10.1 software (FlowJo, Ashland, OR, USA).

### Statistical analysis

Error bars represent standard deviations from the mean of at least three replicates. Two-tailed unpaired Student’s *t* tests were used to compare two groups. *P* values less than or equal to 0.05 were considered to have significance unless specifically described. Flow cytometry data was further analyzed using the Chi Square T(X) test developed by Roederer et al. [45].

## SUPPLEMENTARY MATERIALS



## Abbreviations

cAMP: Cyclic AMP
CRT: Cisplatin and radiotherapy
DMSO: Dimethyl sulfoxide
EDTA: Ethylenediaminetetraacetic acid
ERK: Extracellular signal receptor kinase
HPV: Human Papillomavirus
IFNγ: Interferon gamma
MEK: Mitogen-activated ERK-activating kinase
MOE: Mouse Oropharyngeal Epithelial
OPC: Oropharyngeal cancer
OPSCC: oropharyngeal squamous cell carcinoma
PBS: Phosphate Buffered Saline
PD-1: Programmed cell death protein 1
PD-L1/2: Programmed death ligand 1/2
PI3K: Phosphoinositol-3-Kinase
PTX: Pertussis toxin
SDS-PAGE: Sodium Dodecylsulfate Polyacrylamide Gel Electrophoresis
TBST: Tris Buffered Saline with Tween
