# Supplementary material for: Lactate induces PD-L1 in HRASG12V-positive oropharyngeal squamous cell carcinoma
Source: Oncotarget. 2020 Apr 28;11(17):1493–504. doi: 10.18632/oncotarget.27348 (PMC7197448; doi:10.18632/oncotarget.27348)
Supplement: Supplementary file 1 [file oncotarget-11-1493-s001.pdf]

Lactate induces PD-L1 in HRAS^{G12V}-positive oropharyngeal squamous cell carcinoma

SUPPLEMENTARY MATERIALS

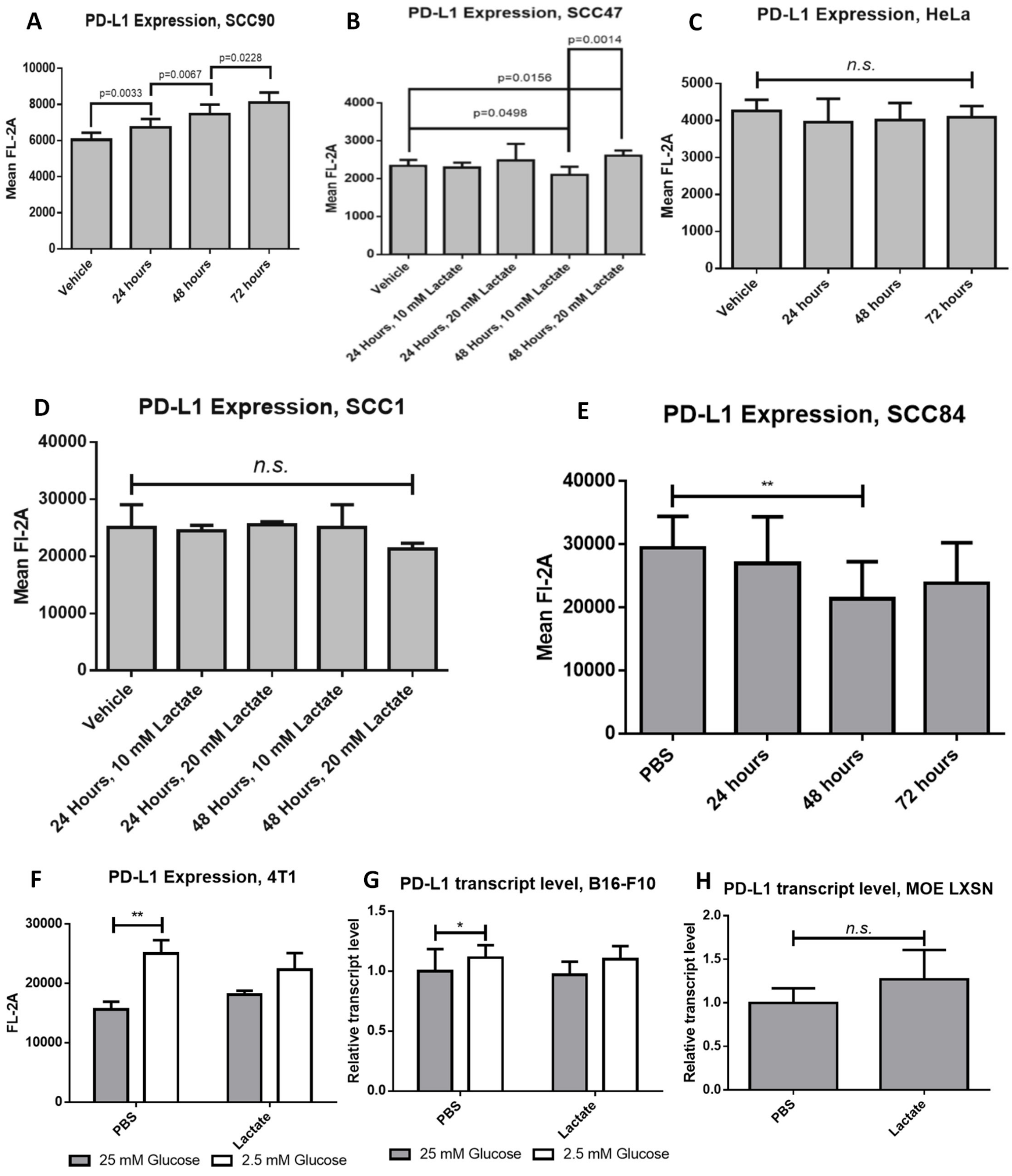

Supplementary Figure 1: Lactate treatment does not increase PD-L1 expression in all cancer lines. (**A**) Flow cytometry data of UPCI:SCC90 (HPV16-positive OPSCC) cells incubated with 10 mM sodium lactate for 24, 48, or 72 hours. (**B**) Flow cytometry analysis of UM-SCC47 (HPV16-positive OPSCC) cells treated with either vehicle or lactate as indicated. (**C**) Flow cytometry analysis of HeLa (HPV18-positive cervical cancer) cells treated with either vehicle or lactate as indicated. (**D**) Flow cytometry analysis of UM-SCC1 (HPV-negative OPSCC) cells treated with either vehicle or lactate. (**E**) Flow cytometry analysis of UM-SCC84 (HPV-negative OPSCC) cells treated with either vehicle or lactate. (**F**) Flow cytometry analysis of 4T1 mammary cancer cells treated with either vehicle or lactate in media containing either 25 mM or 2.5 mM glucose. (**G**) RT-qPCR analysis of B16-F10 melanoma cells treated with either PBS or 10 mM lactate in media containing either 25 mM or 2.5 mM glucose. (**H**) RT-qPCR analysis of MOE LXSN cells treated with either PBS or 10 mM lactate. $^{**}p < 0.01$.
